# Feasibility of Live-Performed Music Therapy for Extremely and Very Preterm Infants in a Tertiary NICU

**DOI:** 10.3389/fped.2020.581372

**Published:** 2020-10-16

**Authors:** Nienke H. van Dokkum, Artur C. Jaschke, Anne-Greet Ravensbergen, Sijmen A. Reijneveld, Laurien Hakvoort, Marlou L. A. de Kroon, Arend F. Bos

**Affiliations:** ^1^Division of Neonatology, Department of Pediatrics, Beatrix Children's Hospital, University Medical Center Groningen, University of Groningen, Groningen, Netherlands; ^2^Department of Health Sciences, University Medical Center Groningen, University of Groningen, Groningen, Netherlands; ^3^Department of Music Therapy, ArtEZ University of the Arts, Enschede, Netherlands

**Keywords:** music therapy, extremely preterm infants, comfort, feasibility study, parental involvement

## Abstract

**Objective:** We aimed to investigate the feasibility of live-performed music therapy for extremely and very preterm infants admitted to the neonatal intensive care unit (NICU), and their parents, starting the 1st−2nd week after birth. They may benefit from live-performed music therapy as comforting non-pharmacological intervention.

**Study Design:** We included infants born before 30 weeks' gestation in a single center NICU study. Live-performed music therapy was provided three times per week, tailored to the infant's medical condition. Parents were actively involved. Feasibility was determined as a combination of participation, drop-out, overstimulation (based on COMFORT-Neo scores), and evaluations of the intervention by parents and nurses (using a questionnaire on perceived effects on the parent, their infant and the NICU sound environment).

**Results:** We included 18 infants (90% participation rate), with a gestational age of median 27 weeks (IQR 26–28 weeks), 61% males. One infant (5.6%) dropped-out. Differences of COMFORT-Neo scores during and after sessions compared with before sessions were non-significant; overstimulation by music therapy did not occur. Parents reported high satisfaction (highest score possible of 7) with the interventions and reported improvements in both infant and their own respiratory rates. Nurses also reported high satisfaction with the intervention and perceived a quieter NICU sound environment during and after sessions.

**Conclusion:** Live-performed music therapy for extremely and very preterm infants is feasible and well-tolerated, and is experienced as an added value to developmental care. Future studies should assess both short-term and long-term effects, to determine whether this intervention should be part of routine care at the NICU and whether it is most beneficial to start shortly after birth.

## Introduction

The first 1,000 days from conception onwards are among the most important periods in the development of an individual. An infant's brain is rapidly developing and is extremely sensitive to environmental influences (1). For preterm infants, born before 37 weeks' gestational age, and specifically those born extremely to very preterm (i.e., before 30 weeks' gestation), the period that should have been spent in the womb is interrupted too early. Preterm infants are subsequently admitted to the Neonatal Intensive Care Unit (NICU), where they face multiple challenges. These challenges include stress caused by physical and sensorial influences as well as maternal separation (2). The NICU is not only stressful for preterm infants, but also for their parents (3). Therefore, parent-infant interactions may be less positive and thus less likely to reduce the infants' stress (4).

Over the past decades, music in medicine has received increasing attention, but specific knowledge on the effects of music in neonatal medicine is still scarce. Many of the performed studies on music in the NICU report on the use of recorded music (5), mainly focusing on moderately and late preterm infants (i.e., with a gestational age of >32 weeks) (5, 6). Several studies suggest, however, that live-performed music therapy is more effective (7–9). Music therapy regards the use of live-performed musical interventions by a certified music therapist, and may be more effective because it is tailored to the immediate needs of the infant. Moreover, music therapy enables active involvement of parents. Studies investigating maternal involvement report that music therapy results in reduced maternal anxiety (6, 7).

The feasibility of music therapy and its effects are still largely unknown for preterm infants born before 30 weeks' gestational age, because these infants have not been included in many studies. However, particularly these extremely and very preterm infants experience considerable stress and pain, for which comforting non-pharmacological interventions may be valuable. Therefore, extremely preterm infants may benefit from music therapy, which could be most beneficial if started as early as possible. Research suggest that using music based interventions synchronously to neural development supports brain development through auditory priming and lies at the core of neural plasticity (10, 11). Stimulating the developing neural circuitry with carefully selected and tailored musical notes supports synaptogenesis and connectivity early on (10). Moreover, music reinforces positive behavioral adjustment after stressful events for preterm infants (11). Thus, a start as early as possible may be justified. Therefore, we aimed to investigate the feasibility of music therapy for extremely preterm infants admitted to the NICU, and their parents, from the 1st to 2nd week after birth onwards.

## Methods

### Setting and Population

We included infants born before 30 weeks of gestation and admitted to the tertiary-level NICU of the University Medical Center Groningen (NL) between August 2019 and January 2020. Our choice to include infants born before 30 weeks of gestation was based on the Dutch setting, where criteria to be transferred to a high care ward in a regional hospital (that is when intensive care treatment is no longer a necessity), are a postmenstrual age of 32 weeks or more and a body weight of 1,200 g or more. For two of our regional hospitals, these criteria were 30 weeks and 1,000 g. This allowed us to provide enough sessions of live-performed music therapy during the infants' stay at our NICU to answer our research questions. Parents of eligible infants were approached during the 1st week after birth, and infants were included in the study after both parents provided written informed consent. Infants whose parents did not speak or understand Dutch were not eligible for inclusion. In the timeframe of our study, we had no missing inclusions due to language barriers. The study was approved by the local ethical review board (METc 2019/093) and was registered online (ISRCTN94562698).

Our NICU follows developmental care principles. All medical staff, both nurses and neonatologists, have been trained in the Family and Infant Neurodevelopmental Education (FINE) program, which is based on NIDCAP (Newborn Individualized Developmental Care and Assessment Program) principles.

### Measures and Procedure

#### Music Therapy

Music therapy was provided by a specialized and certified neonatal music therapist. Music was played after opening one or two doors of the incubator. The distance between incubators was ~1.5–2 m. Doors of the other incubators remained closed. To optimize music therapy for preterm infants, it is suggested that a volume between 45 and 60 decibels (dBs) should be achieved (12). A sound meter was placed in the incubator to monitor dBs, always providing levels between 40 and 65 dBs. This level of dBs is sufficient for music therapy provided to the individual infant, but too soft for other infants to hear.

The music therapy was aimed at relaxation, to try to minimize effects of pain and stress during NICU stay, by particularly following respiratory patterns in the improvised musical interventions. Before the music therapy sessions commenced, the music therapist consulted parents and nurses to inquire specific information on the infant's behavioral state and medical condition. It was our distinct intention to actively involve parents in the music therapy sessions. Parental expectations were managed through detailed information on the sessions and value of music therapy for their infant. As the risk of overstimulating an infant this age may grow after a short time, the music therapist provided each infant with approximately three sessions per week of roughly 15 min of music therapy. Infants received individually improvised music interventions using the Remo Lullaby Ocean Disc, guitar-arpeggios or voice interventions, based on the “rhythm, breath, and lullaby” method as developed by Loewy (8).

To prevent overstimulation, we used maximum one instrument (either the Ocean Disc or guitar) and voice in each music therapy session. During the first two to four sessions, the Ocean Disc was the preferred instrument. This musical instrument is a round disc with small metal beads inside, that replicates the “whoosh” -like timbre of the placenta (7). From the third music therapy session onwards the music therapist collaborated with parents to incorporate song-of-kin (8). Song-of-kin concerns recomposing parental favorite music into a lullaby for the infant. Parents are stimulated to provide this song-of-kin to their infant to empower them in their role as caregivers. If parents did not provide a song-of-kin, the music therapist used the melody of “Twinkle Twinkle Little Star,” since previous research demonstrated good results of this melody in older childer and this melody is familiar to many parents (7, 8). We introcuced the guitar with caution, including ~4 consecutive tones in 6/8 patterns, as to prevent possible overstimulation of the infants by overtones.

The music therapist tailored the interventions to the behavioral state of the infant. When an infant was in quiet sleep, the music therapist played calmly and adjusted the tempo of the music to the infant's breathing pattern. In active sleep, quiet wakefulness or active states, the improvisation was adjusted to the muscle tension and breathing pattern of the infant. Playing was personalized to achieve a calm state, mainly by applying guitar or voice. When infants were tense or cried, the music therapist paced with the infant, until a calmer rhythm could be achieved and environmental sounds could be incorporated into the improvisation. In this state, limitation of dynamics, tempo and stimulations from the music were deemed essential. After the sessions, if parents were present, they were provided the opportunity to talk about their experiences and ask questions to the music therapist.

#### Feasibility

The main study endpoint concerned the feasibility of this intervention for extremely preterm infants. We measured feasibility based on the following criteria:

a) More than 50% of parents agree to participate.
b) Less than 20% of infants dropped out.
c) Less than 20% of infants are overstimulated by the intervention.
d) More than 50% of participating parents evaluate the intervention as positive.
e) More than 50% of nurses evaluate the intervention as positive.

Drop-out regarded infants that were too unstable to start with or to continue to participate in the sessions. This stability was determined by the attending neonatologists and nurses.

Overstimulation was determined by the certified music therapist immediately during the sessions. To quantify reactions to music therapy by the infants afterwards, we used the COMFORT-Neo scores (13). The COMFORT-Neo score comprises 6 items that evaluate facial expression, body movements, alertness, calmness, muscle tension and crying (in spontaneously breathing infants) or respiratory responses (in mechanically ventilated infants) (13). Scores range from 6 to 30, with a lower score indicating a more relaxed infant. A cut-off score of 14 or higher is reported to correspond with numeric rating scales (ranging from 1 to 10) for pain or distress above 4, with adequate sensitivity (81%) and specificity (90%) (13). The scores were determined by trained neonatal nurses or the certified music therapist, ~30 min before the start of the sessions, during the session and ~30 min after the session ended.

Regarding evaluation of the therapy, parents and nurses completed a questionnaire developed by the research team, that included questions on satisfaction with the music therapy, involvement in the intervention, and perceived comforting effects on themselves and their infant, as well as perceived effects on the acoustic environment of the NICU. Satisfaction was rated on a 7 point scale (ranging from 1 “not satisfied at all” to 7 “highly satisfied”). Comforting effects were based on questions regarding perceived decreases in respiratory rates and muscle tension. These perceived effects and the acoustic environment were rated on a 5 point scale (ranging from 1 “fast breathing to 5 “slow breathing,” 1 “tense” to 5 “relaxed” and 1 “noisy” to 5 “quiet,” respectively). Respiratory rates and muscle tension were evaluated because we hypothesized that these are effects of music therapy that could be perceived by parents and professionals. The acoustic environment was evaluated, because we hypothesized that music therapy would decrease the noise levels at the ward, even though our aim was not to provide environmental music therapy. We already had a protocol in place aimed to decrease noise levels at our NICU, and we use a Variphone Sound Ear indicating loud noises (14), but we believed that music therapy could contribute to an even quieter acoustic environment. As final part of the questionnaire, parents and nurses could described their experiences with the music therapy. Nurses completed the questionnaire during the study period. Parents completed the questionnaire once their infant had completed the 3 weeks' music therapy intervention or was discharged to a High-Care/Medium-Care unit.

### Statistical Analysis

First, we determined participant characteristics using descriptive statistics. Second, we assessed the feasibility of music therapy based on (1) participation rate, (2) drop-out percentage, (3) overstimulation, (4) evaluation of the intervention by parents, and (5) evaluation of the intervention by nurses. Participation rate and drop-out were assessed using descriptive statistics. We assessed overstimulation using the COMFORT-Neo scores, and tested the differences of these scores between three pairs of observations using the Wilcoxon signed rank test: (1) observations during the sessions and observations before the sessions, (2) observations after the sessions and observations before the sessions, and (3) observations after the sessions and observations during the sessions. Parents' and nurses' experiences with the music therapy were assessed using descriptive statistics. We did not perform statistical tests on the evaluation questionnaire completed by parents and nurses regarding satisfaction with the music therapy, perceived effects on respiratory rate, muscle tension and acoustic environment of the NICU, because of the fairly small numbers included in this pilot study. Analyses were performed using SPSS version 26.0 (IBM Statistics, NY, USA). *P* < 0.05 were considered statistically significant.

## Results

### Participant Characteristics, Participation Rate and Drop-Out Percentage

In total, 26 infants were eligible to participate in the study. Of these, three deceased before participation could be asked, and three were not approached for permission because of logistical reasons. Parents of the remaining 20 infants were asked to participate. Parents of two infants declined to participate, either for all research activities or, specifically for this study, due to worries for overstimulation of their infant. Therefore, 18 infants were included in the study (90% participation rate).

Of the 18 included infants, median gestational age at birth was 27 weeks (Interquartile Range [IQR] 26–28 weeks), median birth weight was 1,003 g (IQR 798–1,136 g). Eleven infants (61%) were males, all were singletons. Infants received their first music therapy session at median postnatal day 16 (IQR 12–22), with a postmenstrual age of median 30 weeks (IQR 28–31 weeks). The 18 infants received 81 music therapy sessions in total. During the study period, two of the participating infants deceased.

One participating infant (gestational age at birth 24 weeks) was initially, at a postmenstrual age of 28 weeks, rated as being too unstable to be offered music therapy sessions (i.e., a drop-out), because of frequent apnea, bradycardias and saturation dips during several procedures. The drop-out percentage in this study was therefore one out of 18 (5.6%). Attending neonatologists and nurses rated this infant as being sufficiently stable several weeks later (30 weeks' postmenstrual age) and the infant did receive several sessions of music therapy and was included in the study.

### Overstimulation

**Figure 1:**
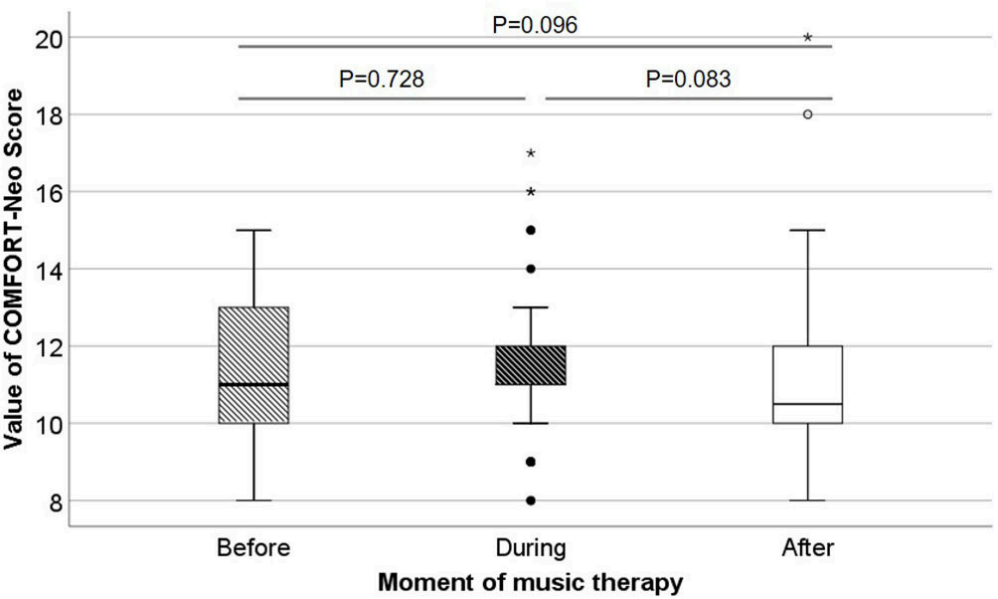
COMFORT-Neo scores before, during and after music therapy. In this box whisker plots, the boxes represent the interquartile ranges, the whiskers represent the range of all ages and the dots and stars represent outliers.

The music therapist observed the infants during the music therapy sessions specifically searching for signs of overstimulation, but did not detect any signs, she specifically did not observe any desaturations. Regarding the responses of the infants, we present the distribution of COMFORT-Neo scores before, during and after the sessions in Figure 1. Before the sessions, median scores were 11 (IQR 10–13), during the music therapy sessions, median scores were 11 (IQR 10–12) and after the sessions median scores were 10.5 (IQR 10–12). Scores below the cut-off score of 14 all remained below 14. If 14+, this indicates pain or distress in preterm infants. COMFORT-Neo scores did not statistically significantly differ before, during and after sessions.

### Parents' Evaluation of Music Therapy

The parental evaluation questionnaire was completed by parents of 11 individual infants, only one parent per infant completed the questionnaire. Five of the respondents were present during one or more music therapy sessions. All respondents who were present during at least one music therapy session were very satisfied and rated the sessions with the highest possible score of 7. Parents reported that music therapy made them feel relaxed. Parents also perceived that their infants displayed signs of comfort during and after music therapy sessions. Parents did not perceive changes in the acoustic environment of the NICU. All parents reported to feel very involved in the music therapy sessions. One of the parents strikingly labeled the music therapy “a very valuable addition to neonatal care and future development.”

### Nurses' Evaluation of Music Therapy

**Figure 2:**
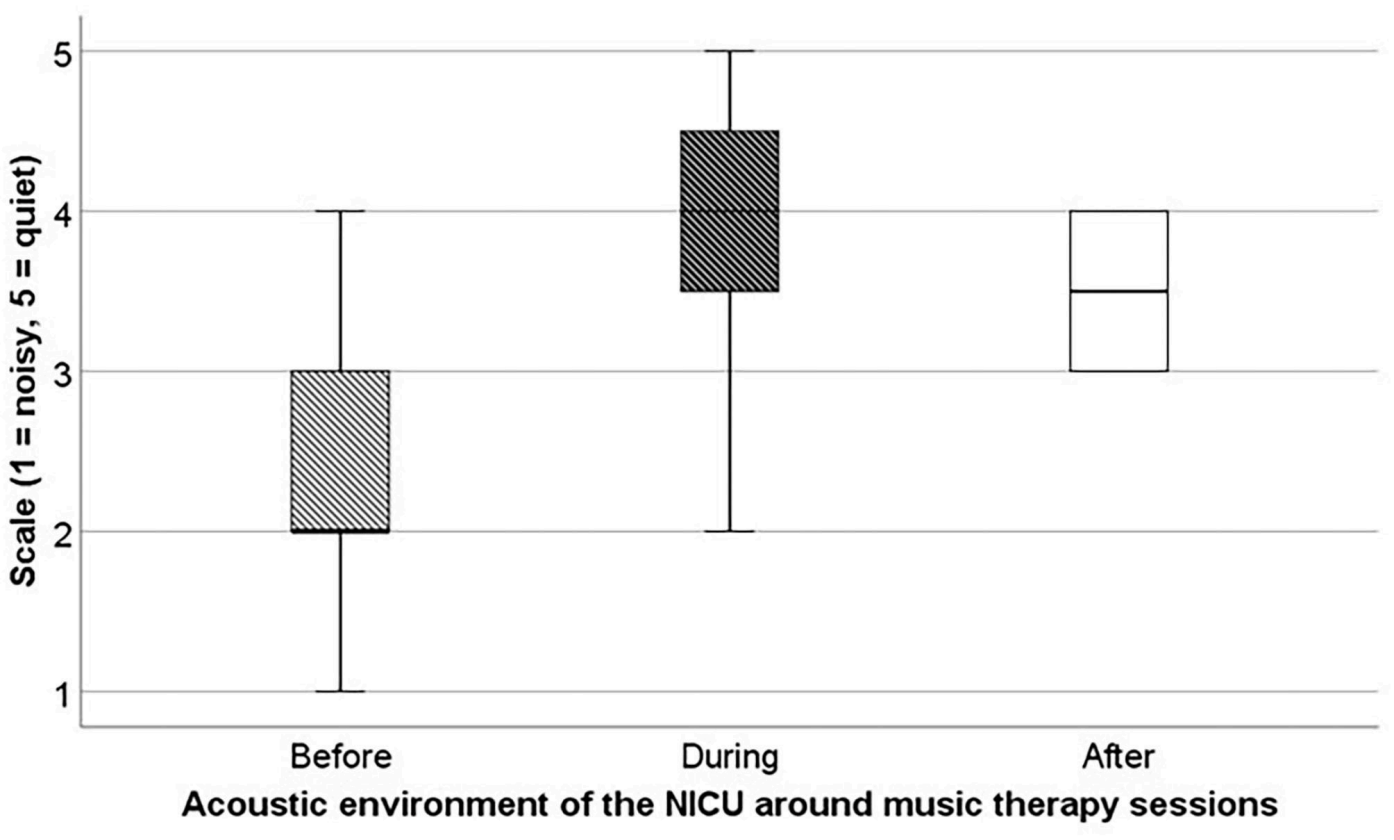
Average acoustic environment of the NICU according to nurses before, during and after music therapy. Acoustic environment of the NICU was scored on a scale from 1 “noisy” to 5 “quiet.” In this box whisker plots, the boxes represent the interquartile ranges, the whiskers represent the range of all ages.

The evaluation questionnaire was completed by 15 trained neonatal nurses, of which 12 attended at least one music therapy session and the others were at the unit, but not at the incubator side during sessions. All nurses were satisfied with the music therapy sessions on the neonatal unit, with a mean score of 5.6 (on a scale from 1 to 7). Only one nurse reported an improvement in her own respiratory rate and muscle tone. Regarding the acoustic environment of the NICU, 75% perceived slight improvements in the noise levels (Figure 2).

## Discussion

The aim of this study was to investigate the feasibility of music therapy for extremely and very preterm infants admitted to the NICU, and their parents. The participation rate of parents was high and the drop-out rate of infants was fairly low. Remarkably, most of these extremely and very preterm infants were sufficiently stable to participate in the music therapy sessions. Moreover, the certified music therapist did not observe overstimulation of the infants, and we found no overstimulation of the infants' reactions as measured by COMFORT-Neo scores. Finally, both parents and nurses evaluated the music therapy sessions as positive. Therefore, we consider live-performed music therapy by a certified and specifically trained neonatal music therapist feasible for extremely preterm infants.

We found that infants were generally stable enough to participate in music therapy and were not overstimulated by sessions. This reflects the scarce literature, existing of only one other feasibility study, reporting that extremely preterm infants tolerate live-music quite well (15). Of note, that study did not regard music therapy, but rather a musical performance, as it was not individualized to infants. In infants born after 32 weeks' gestation, music therapy, as opposed to recorded music or live performance, has been reported to have positive effects on physiological states (i.e., lowered heart rate, lowered respiratory rate and improved oxygenation, thus well-tolerated by these infants), neurobehavioral profiles and feeding outcomes (16). Future research should focus on the effects of music therapy in extremely preterm infants on vital parameters, physiological stability and stress levels, because effects of this intervention show promising results in slightly older infants (7, 9, 17).

Our results also demonstrated that both parents and nurses evaluate the music therapy as very positive. Parents reported subjective effects on their infants' and their own breathing and muscle tone. In our study, only a limited amount of parents were present. This can be explained by the fact that we have an open-bay unit, where parents are already present for several hours a day, including at least an hour of Kangaroo care. Parental presence and Kangaroo care are part of our developmental care program. Mothers may still be ill or recovering from preterm labor and parents may therefore choose not to be present during the music therapy sessions. Moreover, the availability of our music therapist was limited, because she was only present during mornings. In contrast to parents, nurses reported effects on the perception of acoustic environment of the NICU. We did not find evidence regarding evaluation of music therapy by parents, apart from reduced maternal anxiety (6). One study investigated nurses' expectations of music use in preterm infants and reported expected positive effects on hospitalized infants, as well as parents and medical staff (18). However, full evaluations of music therapy in the NICU by neonatal nurses are not available. Future studies should focus on evaluation of music therapy by parents and nurses incorporating their perception on the effects of music therapy on the infants, as well as bonding and parent-infant interactions, to improve active involvement of parents.

We consider music therapy by a certified and well-trained neonatal music therapist to be feasible for extremely and very preterm infants admitted to a tertiary NICU as it creates a possible link to *in-utero* exposure to certain musical sounds (i.e., rhythmic heartbeat, maternal voice, or music listened to by the mother). Music therapy, therefore, may be beneficial for the tiniest infants already from the very beginning, since these infants are already able to respond adequately to sounds (19). Studies on music in preterm infants report that music may enhance formation and maturation of structural brain networks involved in cognition and emotion regulation (20, 21). It is therefore hypothesized that early auditory experiences are associated with structural brain development (10). In music therapy, the music therapist tailors the musical intervention to specifically meet the needs of the individual infant at that particular moment. These music therapy interventions may be used in several facets during NICU stay, to offer a positive auditory stimulation which could promote brain development (5), but also to comfort the infant during painful procedures (17) and promote sleep (22). Therefore, we believe that music therapy should be further evaluated as a promising valuable part of standard care at NICUs around the world. Fortunately, such efforts are already being made (23).

## Strengths and Limitations

To our knowledge, this is the first study to investigate the feasibility of live-performed music therapy for extremely and very preterm infants. Major strengths of our study include the structured manner in which we offered the music therapy sessions, the active involvement of parents in the sessions, and the evaluation of the perception of parents and nurses. One of the limitations of our study concerns the small sample size, especially regarding the evaluations of parents and nurses. Additionally, only a few parents were present during the music therapy sessions. Being a feasibility study, however, we believe that the sample size is adequate. Second, the COMFORT-Neo scores were assessed by different professionals, either the certified music therapist or trained neonatal nurses. Because the inter-rater reliability of the COMFORT-Neo scores is relatively high, with a kappa value of 0.79 (13), we believe that these different assessors did not have major impacts on our results. Nonetheless, assessment of the COMFORT-Neo by the music therapist may have introduced bias toward lower scores. Finally, the nurses and music therapist were aware of the study purpose and this lack of blinding might have influenced a positive outcome.

## Conclusion

Live-performed music therapy is a feasible intervention for extremely and very preterm infants. This study indicates that it is well-tolerated and does not overstimulate the infants. Parents and nurses evaluate music therapy as an added value to developmental care during NICU stay. Future studies should focus on the effects of music therapy on physiological stability and neurodevelopment both short-term and long-term, to determine whether this intervention could be part of routine care at the NICU. Moreover, future studies should focus on experiences of parents and nurses with music therapy in the NICU to stimulate active involvement.

## Data Availability Statement

The raw data supporting the conclusions of this article will be made available by the authors, without undue reservation.

## Ethics Statement

The studies involving human participants were reviewed and approved by Medical Ethical Review Board of the University of Groningen. Written informed consent to participate in this study was provided by the participants' legal guardian/next of kin.

## Author Contributions

ND conceptualized and designed the study, collected and analyzed the data, and drafted the first and final manuscript. AJ contributed to the study design, supervised the execution of the music therapy intervention, contributed to the interpretation of the findings, and critically reviewed and revised the manuscript. A-GR collected the data as certified neonatal music therapist and critically reviewed and revised the manuscript. SR and MK contributed to the study design and critically reviewed and revised the manuscript. LH supervised the execution of the music therapy intervention and critically reviewed and revised the manuscript. AB conceptualized and designed the study, supervised the collection and analyzes of the data, contributed to the interpretation of the findings, and critically reviewed and revised the manuscript. All authors gave final approval of the version to be published and agreed to be accountable for all aspects of the work.

## Conflict of Interest

The authors declare that the research was conducted in the absence of any commercial or financial relationships that could be construed as a potential conflict of interest.
